# Degenerative pathologies on cortical biopsy, dopaminergic depletion, and shunt efficacy in iNPH

**DOI:** 10.1002/alz.70974

**Published:** 2025-12-09

**Authors:** Byoung Seok Ye, So Hee Park, Seun Jeon, Sungwoo Kang, Se Hoon Kim, Won Seok Chang

**Affiliations:** ^1^ Department of Neurology Yonsei University College of Medicine Seoul Republic of Korea; ^2^ Metabolism‐Dementia Research Institute Yonsei University College of Medicine Seoul Republic of Korea; ^3^ Department of Neurosurgery Yeungnam University College of Medicine Daegu Republic of Korea; ^4^ Department of Neurology Hanyang University College of Medicine Seoul Republic of Korea; ^5^ Department of Pathology Yonsei University College of Medicine Seoul Republic of Korea; ^6^ Department of Neurosurgery Yonsei University College of Medicine Seoul Republic of Korea

**Keywords:** Alzheimer's disease, cortical biopsy, dopamine transporter imaging, idiopathic normal pressure hydrocephalus, ventriculoperitoneal shunting

## Abstract

**INTRODUCTION:**

Coexisting degenerative pathologies may influence the efficacy of ventriculoperitoneal (VP) shunting in idiopathic normal pressure hydrocephalus (iNPH).

**METHODS:**

We evaluated 58 iNPH patients who underwent VP shunting with cortical biopsy assessing pathologies including amyloid beta (Aβ). Concordance between biopsy‐identified Alzheimer's pathology and amyloid positron emission tomography (PET) and the influence of magnetic resonance imaging‐based iNPH indices, dopamine transporter (DAT) imaging, and biopsy pathologies on postoperative outcomes were examined.

**RESULTS:**

Aβ pathology was found in 23 patients (39.7%) and showed high concordance with amyloid PET (95.2%). Although Aβ positivity was associated with poorer 1‐year cognitive outcome, and cognitive improvement reached significance only in Aβ‐negative patients, both Aβ‐positive and Aβ‐negative groups exhibited functional improvement after surgery. Lower anterior striatal DAT uptake was paradoxically associated with better postoperative outcomes, particularly in Aβ‐positive individuals.

**DISCUSSION:**

Cortical biopsy provides information concordant with amyloid PET, and biopsy‐confirmed Alzheimer's pathology and dopaminergic status influence shunt efficacy. VP shunting remains beneficial in iNPH even with degenerative comorbidities.

**Highlights:**

- Cortical biopsy findings showed high concordance with amyloid PET in iNPH patients.
- AD pathology was linked to poorer cognitive and functional outcomes after surgery.
- VP shunting remained beneficial even in patients with comorbid AD pathology.
- Lower anterior striatal DAT uptake was paradoxically associated with better outcomes.
- Combined use of biopsy and DAT imaging may aid prognostication in iNPH management.

## 1 BACKGROUND

Idiopathic normal pressure hydrocephalus (iNPH) is a relatively uncommon but clinically significant neurological disorder that predominantly affects older adults. It is characterized by an abnormal accumulation of cerebrospinal fluid (CSF) in the brain's ventricles, leading to a distinctive triad of symptoms: gait disturbances, cognitive impairment, and urinary incontinence.^1^ The prevalence of iNPH increases with age, most commonly affecting individuals over 60. Two recent epidemiological surveys in Sweden reported an iNPH prevalence of 3.7% among 65‐year‐olds, rising to 5.9% and 8.9% in those over 80.^2^, ^3^ Given that degenerative brain diseases such as Alzheimer's disease (AD) and Lewy body disease (LBD) also become more prevalent with age,^4^, ^5^ iNPH is likely to coexist with these conditions. Moreover, iNPH and degenerative diseases may share a pathophysiological link. LBD shares several symptoms with iNPH, including gait disturbances, bradykinesia, and cognitive decline.^6^, ^7^ Additionally, dopaminergic depletion detected through dopamine transporter (DAT) imaging, a key biomarker for LBD, has also been reported in iNPH.^8^, ^9^

Since Hakim and Adams first described iNPH in 1965,^10^ CSF shunting has remained the primary treatment. Among the available options, ventriculoperitoneal (VP) shunting is the most commonly used and the only treatment with proven efficacy.^4^ The VP shunt redirects excess CSF from the brain's ventricles to the peritoneal cavity, relieving iNPH‐related symptoms. However, post‐surgical outcomes in iNPH range from significant improvement to only partial symptom relief.^11^, ^12^ A minimally invasive brain tissue biopsy during VP shunt surgery enables the identification of degenerative brain pathology.^13^ We hypothesized that coexisting degenerative pathology would significantly influence the therapeutic response to VP shunting in iNPH patients. For instance, the presence of AD pathology may lead to poorer outcomes or a diminished clinical response to shunt surgery.^14^, ^15^

This study aimed to assess the concordance between AD biomarkers detected via amyloid positron emission tomography (PET) and degenerative pathology identified through biopsy during VP shunt surgery. Additionally, we investigated whether degenerative pathologies identified through biopsy, along with DAT imaging and magnetic resonance imaging (MRI) indices of iNPH, affected postoperative outcomes following VP shunt surgery.

## 2 METHODS

### 2.1 Study participants and clinical diagnoses

**FIGURE 1 alz70974-fig-0001:**
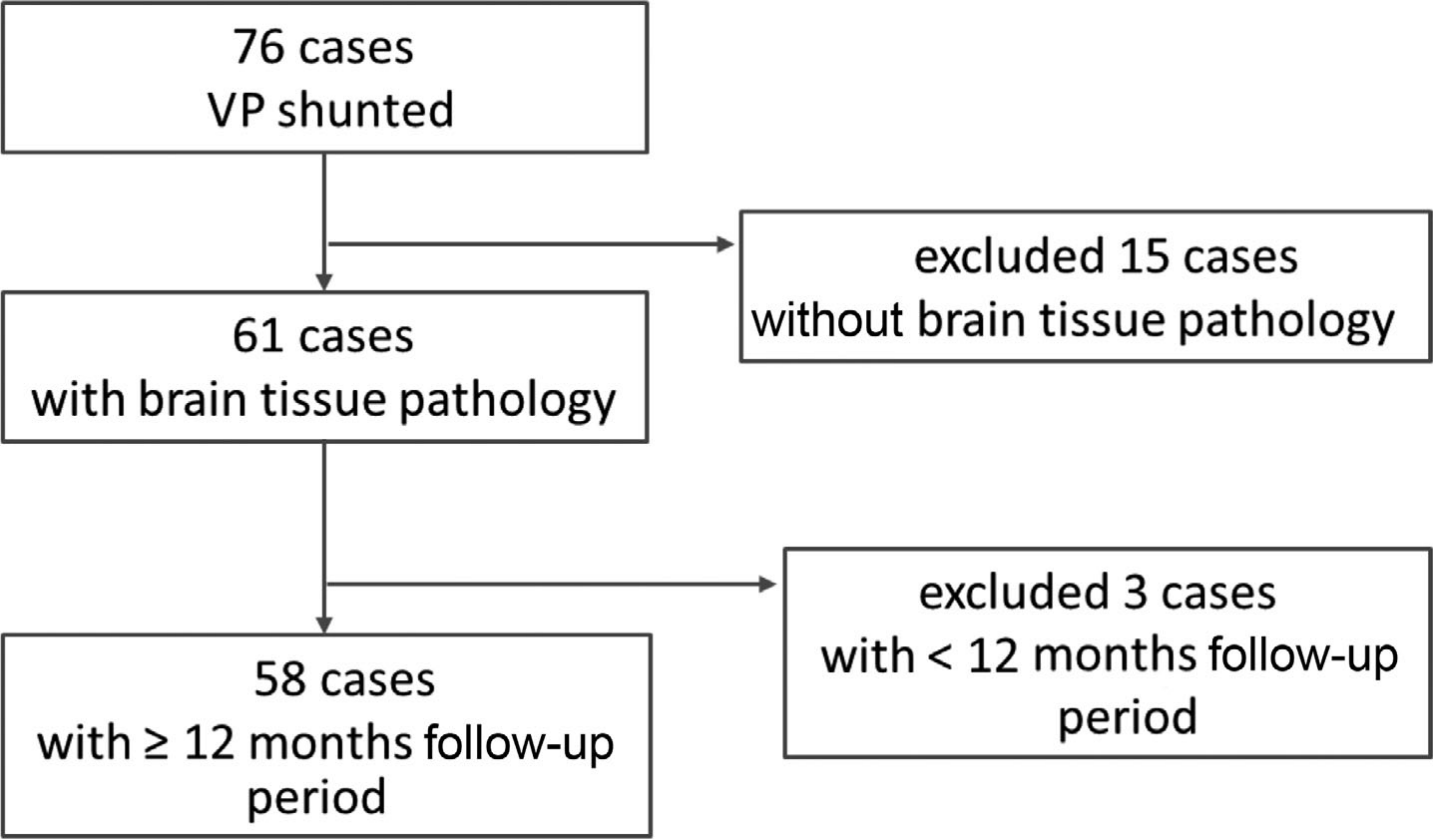
Flowchart of patient selection. Among 76 patients who underwent ventriculoperitoneal (VP) shunting, 15 were excluded due to lack of cortical biopsy pathology. Of the remaining 61 cases with available cortical biopsy data, three were further excluded due to a follow‐up period of less than 12 months. A total of 58 patients with cortical biopsy pathology and a follow‐up period of 12 months or longer were included in the analysis.

We conducted a retrospective study spanning a 5‐year period, from April 2017 to March 2022, focusing on patients with iNPH who underwent VP shunt surgery at a single center. Patient demographics, neurological and neuropsychological assessments conducted before and after a large‐volume lumbar tap, operative notes, histopathology reports, and follow‐up clinic charts were reviewed. During the study period, a total of 76 patients underwent VP shunt surgery. Following the exclusion of those where immunohistochemical stains were not performed on brain tissue and those with a follow‐up duration of less than 12 months, a cohort of 58 patients (male 25, female 33) was selected for analysis (Figure 1).

**RESEARCH IN CONTEXT**

1. **Systematic review**: We reviewed the literature on coexisting neurodegenerative pathology in iNPH, including studies using cortical biopsy, amyloid PET, and DAT imaging. A comprehensive search on PubMed was conducted through March 2024. Prior studies reported Alzheimer's pathology in up to 50% of iNPH cases, but the prognostic impact of these comorbidities remains controversial. Few studies have directly compared biopsy findings and amyloid PET or integrated multimodal imaging with long‐term clinical outcomes.
2. **Interpretation**: Our findings demonstrate that cortical biopsy reliably reflects amyloid PET status and identifies substantial Alzheimer's pathology in iNPH patients. Although Aβ and pTau were associated with worse cognitive and functional outcomes, VP shunting still resulted in overall clinical improvement. Notably, reduced anterior striatal DAT uptake was paradoxically associated with better outcomes, suggesting that subcortical dopaminergic dysfunction may identify a subgroup more responsive to CSF diversion.
3. **Future directions**: Future prospective studies with larger samples should further validate the prognostic utility of cortical biopsy and dopaminergic imaging in iNPH. Longitudinal analyses comparing pre‐ and post‐shunt DAT imaging may clarify mechanisms underlying response to treatment. Integration of these biomarkers into iNPH diagnostic criteria could facilitate individualized treatment planning.

Seven patients had clinical diagnoses of iNPH only, while 51 patients had coexisting diagnoses, including AD (*N* = 17), dementia with Lewy bodies (DLB, *N* = 29), possible LBD (pLBD, *N* = 12), and vascular cognitive impairment (VCI, *N* = 5). The diagnosis of AD was based on the criteria established by the National Institute of Neurological Communicative Disorders and Stroke and the Alzheimer's Disease and Related Disorder Association (NINCDS‐ADRDA).^16^ Since patients with iNPH can also present with parkinsonism^6^, ^7^ and abnormal DAT imaging,^8^, ^9^ the diagnosis of DLB was made only when patients exhibited at least one of the core clinical features of DLB, specifically cognitive fluctuations or visual hallucinations.^17^ Therefore, patients with dementia, parkinsonism, and abnormal DAT‐PET findings, or those with dementia, parkinsonism, and rapid eye movement sleep behavioral disorder (RBD), met the 2017 McKeith criteria for probable DLB.^17^ However, we did not classify these patients as DLB in this study. In addition, patients who exhibited parkinsonism, mild fluctuations, or visual illusions that did not fulfill the diagnostic criteria for DLB were categorized as pLBD. The diagnosis of VCI was made if patients had brain MRI demonstrated Fazeka grade 3 ischemic changes^18^ or when a temporal relationship between a cerebrovascular event and subsequent cognitive deterioration was confirmed.^19^ Descriptions of individual cases are provided in Table S1.[Fig alz70974-fig-0001]

### 2.2 Imaging analyses

MRI scans were performed in 55 (94.8%) patients to measure iNPH‐related MRI indices, including the callosal angle, Evans index, ventriculomegaly score, dilated Sylvian fissure score, tight high convexity score, acute callosal angle score, focal sulcal dilation score, and the disproportionately enlarged subarachnoid space hydrocephalus (DESH) scale, as described previously.^20^, ^21^, ^22^ Twenty‐one of 58 (36.2%) patients underwent ^18^F‐Florbetaben (FBB) PET to evaluate cerebral amyloid deposition. Both visual assessment and automated quantification were performed, as previously described.^23^, ^24^ Quantification used the cerebellar gray matter as the reference region, and global standardized uptake value ratio (SUVR) values were calculated as the volume‐weighted cortical average across frontal, anterior/posterior cingulate, lateral parietal, and lateral temporal regions of interest. Quantification analysis was available for 14 patients. FBB‐PET was considered positive if the global FBB‐PET SUVR exceeded 1.478.^25^ For the remaining seven patients, in whom quantification could not be performed due to the absence of a three‐dimensional T1‐weighted turbo‐echo sequence, FBB‐PET positivity was determined based on visual assessment. DAT‐PET scans were performed in 34 of 58 patients (58.6%) using ^18^F‐N‐(3‐fluoropropyl)‐2β‐carboxymethoxy‐3β‐(4‐iodophenyl) nortropane (FP‐CIT). However, due to difficulties in image preprocessing, DAT‐PET images from six patients were excluded from further analysis. SUVR maps of delayed‐phase FP‐CIT PET images were generated using the occipital white matter as the reference region. Regional DAT uptake was measured in the anterior/posterior caudate and anterior/posterior putamen, as described in our previous study.^26^ Abnormal DAT uptake was defined as striatal SUVR values more than −1.5 standard deviations below those of healthy controls in our previous study.^26^

### 2.3 Preoperative assessment

The evaluation for iNPH was performed by a neurologist (Y.B.S.) and a neurosurgeon (C.W.S.). A large‐volume spinal tap of approximately 50cc was performed to confirm improvement in gait and cognitive symptoms. Symptom changes were evaluated using objective measures, including the Korean Mini‐Mental State Examination (K‐MMSE), the Timed Up and Go (TUG) test, and the 10‐m‐walk test, administered before and after the tap. For patients unable to complete these tests due to their condition, clinical improvement was assessed based on caregiver‐reported changes in symptoms.

### 2.4 Operative procedures and histological procedures

Surgical intervention was considered for patients who showed symptom improvement following clinical assessments. Cortical biopsy was performed as part of the routine clinical procedure during the study period, and this was included in the explanation and consent process provided to patients and their legal guardians. Due to the retrospective nature of this study, the requirement to obtain informed consent to participate in this study was waived. The study was approved by the Institutional Review Board of Severance Hospital. A VP shunt was placed via a right frontal approach, and the peritoneal catheter was inserted through an abdominal incision. Following the creation of a burr hole and dura incision at the right Kocher's point (3 cm lateral to the midline and 1 cm anterior to the coronal suture), a cortical brain biopsy was performed. A cortical area free of overlapping blood vessels was identified, and multiple 3 × 3 × 3‐mm cortical tissue samples were obtained and preserved in neutral buffered formalin. A Medtronic Strata Delta valve was used, and the initial shunt pressure setting was approximately 20 mmH_2_O lower than the patient's ventricular opening pressure.

The brain specimens were paraffin‐embedded and sectioned following fixation. Consecutive sections were then subjected to hematoxylin and eosin (H&E) staining and immunohistochemical staining for markers including Aβ, AT8 (phosphorylated tau, pTau), α‐synuclein, TAR DNA‐binding protein 43 (TDP‐43), and glial fibrillary acidic protein (GFAP). Immunostaining was performed using the BenchMark ULTRA PLUS system (Roche Diagnostics). We performed immunohistochemical staining with both negative and positive control slides. For Aβ, an anti‐Aβ (17‐24) antibody from BioLegend was used at a dilution of 1:500, with standard epitope retrieval (64 min) and primary antibody incubation for 32 min. For pTau (AT8), an antibody from Thermo Fisher Scientific (Catalog No.: MN1020) was used at a 1:40 dilution with standard epitope retrieval (64 min) and 32 min of incubation. For α‐synuclein, a primary antibody from Abcam (Catalog No.: ab59264) was used at a 1:500 dilution with mild epitope retrieval (32 min) and 32 min of incubation. TDP‐43 immunostaining was performed using an antibody from Cosmo Bio (Catalog No.: TIP‐PTD‐M01, clone 11‐9) at a 1:3000 dilution after mild epitope retrieval (32 min). For GFAP, a polyclonal antibody from DAKO (Catalog No.: 6F2) was used at a 1:2000 dilution without epitope retrieval, with a primary antibody incubation time of 16 min. All histologic assessments were performed by a neuropathologist (K.S.H.) who was blinded to clinical information.

### 2.5 Postoperative management

Postoperative follow‐up evaluations included regular computed tomography (CT) scans and clinical assessments of patients’ symptoms. Shunt valve pressure adjustments were individually tailored based on these comprehensive evaluations. Patient outcomes were assessed annually using the Modified Rankin Scale (mRS). Additionally, for patients with a preoperative K‐MMSE score between 10 and 26, the K‐MMSE was administered annually to monitor cognitive function and track changes over time.

### 2.6 Statistical analysis

Statistical analyses were performed with R software (version 4.2.0.; https://cran.r‐project.org) and SPSS version 27.0 (IBM Corp., Armonk, NY, USA). To compare clinical characteristics, biopsy pathology, imaging findings, and diagnostic variables between patients with positive biopsy staining for Aβ (Aβ+) and those without (Aβ−), independent *t* tests and chi‐squared tests were conducted. Linear mixed‐effects models (LMMs) adjusted for age, sex, and education were applied to assess the significance of pre‐ to postoperative changes in MMSE and mRS scores in the total cohort and in the Aβ− and Aβ+ subgroups. To identify predictors of 1‐year and 2‐year postoperative K‐MMSE scores or mRS, general linear models (GLMs) were applied, each using a single predictor among biopsy pathology markers, MRI findings, and DAT uptake values, while controlling for age, sex, education, and baseline K‐MMSE scores or mRS. Among the biopsy markers, Aβ, α‐synuclein, TDP‐43, and pTau were included due to the limited diagnostic specificity of other pathology markers. All statistical analyses were performed using a two‐sided significance level of 0.05.

## 3 RESULTS

### 3.1 Clinical and biopsy pathology characteristics of study participants

The mean age of the cohort was 73.97 ± 8.55 years. The average follow‐up period was 34.71 ± 18.76 months (Table 1). There were 23 (39.7%) patients with positive staining for Aβ. The Aβ+ group tended to be older than the Aβ− group (mean age, 76.52 ± 6.96 vs 72.29 ± 9.16, *p* = 0.064), but the two groups had comparable sex ratio, years of education and follow‐up durations. Fourteen of 23 (60.9%) patients in the Aβ+ group had AD, while three of 35 (8.6%) patients in the Aβ− group did (*p* < 0.001). The proportion of patients diagnosed with DLB tended to be higher in the Aβ+ group than in the Aβ− group (15/23 = 65.2% vs 14/35 = 40.0%, *p* = 0.060). However, the proportions of patients with pLBD and VCI were comparable between the two groups. The Aβ+ group had a higher proportion of *APOE* ε4 carriers than the Aβ− group (6/19 = 31.6% vs 2/30 = 6.7%, *p* = 0.043).

**TABLE 1:** Demographic and clinical characteristics of study participants.

	Overall	Aβ−	Aβ+	*p*‐value
Number	58	35	23	
Age, years	73.97 (8.55)	72.29 (9.16)	76.52 (6.96)	0.064
Female, *N* (%)	33 (56.9%)	20 (57.1%)	13 (56.5%)	0.963
Education, years	10.46 (4.53)	11.00 (4.17)	9.63 (5.02)	0.264
Follow‐up duration	34.71 (18.77)	36.26 (19.17)	32.35 (18.31)	0.443
Clinical diagnosis				
AD	17/58 (29.3%)	3/35 (8.6%)	14/23 (60.9%)	<0.001
DLB	29/58 (50.0%)	14/35 (40.0%)	15/23 (65.2%)	0.060
pLBD	12/58 (20.7%)	8/35 (22.9%)	4/23 (17.4%)	0.746
VCI	5/58 (8.6%)	5/35 (14.3%)	0	0.146
*APOE ε4* carrier^a^	8/49 (16.3%)	2/30 (6.7%)	6/19 (31.6%)	0.043
Biopsy pathology^a^				
α‐synuclein	2/56 (3.6%)	1/35 (2.9%)	1/21 (4.8%)	>0.999
TDP‐43	2/56 (3.6%)	0/35 (0%)	2/21 (9.5%)	0.136
pTau	11/57 (19.3%)	0/35 (0%)	11/22 (50.0%)	<0.001
GFAP	51/56 (91.1%)	31/35 (88.6%)	20/21 (95.2%)	0.640
FBB‐PET^a^				<0.001
Positive	11/21 (52.4%)	0/9 (0%)	11/12 (91.7%)	
Negative	10/21 (47.6%)	9/9 (100%)	1/12 (8.3%)	
DAT‐PET abnormality^a^	30/34 (88.2%)	19/20 (95.0%)	11/14 (78.6%)	0.283
DAT uptake value^b^				
Anterior caudate	3.48 (0.90)	3.46 (0.69)	3.51 (1.14)	0.886
Posterior caudate	2.67 (0.81)	2.78 (0.60)	2.54 (1.03)	0.447
Anterior putamen	4.05 (0.93)	3.91 (0.72)	4.22 (1.15)	0.384
Posterior putamen	3.40 (0.94)	3.31 (0.82)	3.51 (1.10)	0.572
MRI findings^e^				
Callosal angle	72.56 (20.70)	72.24 (23.04)	73.07 (16.88)	0.887
Evans index	0.37 (0.05)	0.38 (0.06)	0.36 (0.04)	0.231
Ventriculomegaly score	1.65 (0.55)	1.67 (0.60)	1.64 (0.49)	0.844
Dilated Sylvian fissure score	1.04 (0.71)	0.94 (0.72)	1.19 (0.70)	0.210
Tight high convexity score	1.06 (0.64)	1.11 (0.66)	1.00 (0.61)	0.556
Acute callosal angle score	1.67 (0.64)	1.64 (0.70)	1.71 (0.56)	0.669
Focal sulcal dilatation score	0.63 (0.70)	0.62 (0.71)	0.64 (0.71)	0.913
DESH scale	6.05 (1.79)	5.97 (2.01)	6.17 (1.41)	0.697
K‐MMSE^c^				
Baseline	17.02 (7.96)	19.15 (7.63)	13.87 (7.51)	0.013
1‐yyear postoperative	18.71 (7.24)	21.12 (5.78)	14.94 (7.84)	0.006
2‐year postoperative	19.13 (8.05)	21.65 (5.80)	15.27 (9.60)	0.031
mRS^d^				
Baseline	3.45 (0.73)	3.29 (0.71)	3.70 (0.70)	0.035
1‐year postoperative	2.67 (0.98)	2.54 (0.95)	2.87 (1.01)	0.217
2‐year postoperative	2.83 (1.03)	2.57 (0.92)	3.21 (1.08)	0.035

*Note*: Data are results of chi‐squared test or independent *t* test as appropriate.

Abbreviations: AD, Alzheimer's disease; *APOE, apolipoprotein*; DAT, dopamine transporter; DESH, disproportionately enlarged subarachnoid space hydrocephalus; DLB, dementia with Lewy bodies; FBB, ^18^F‐Florbetaben; K‐MMSE, Korean‐version Mini‐Mental State Examination; MRI, magnetic resonance imaging; mRS, Modified Rankin Scale; PET, positron emission tomography; pLBD, possible Lewy body disease; TDP‐43, TAR DNA‐binding protein 43; VCI, vascular cognitive impairment.

^a^ Tests were performed in subset of patients, with results presented as N/total number tested (%).

^b^ DAT‐PET imaging analyses were performed in 16 patients in the Aβ− group and 13 in the Aβ+ group.

^c^ Baseline K‐MMSE was not available for one patient in the Aβ− group. The 1‐year postoperative K‐MMSE was unavailable for 10 patients in the Aβ− group and seven in the Aβ+ group, while the 2‐year postoperative K‐MMSE was unavailable for 12 patients in the Aβ− group and eight in the Aβ+ group.

^d^ Baseline and 1‐year postoperative mRS were assessed in all patients; however 2‐year postoperative mRS data were unavailable for seven patients in the Aβ− group and four in the Aβ+ group.

^e^ MRI was not performed in two patients in the Aβ− group and one in the Aβ+ group.

**FIGURE 2:**
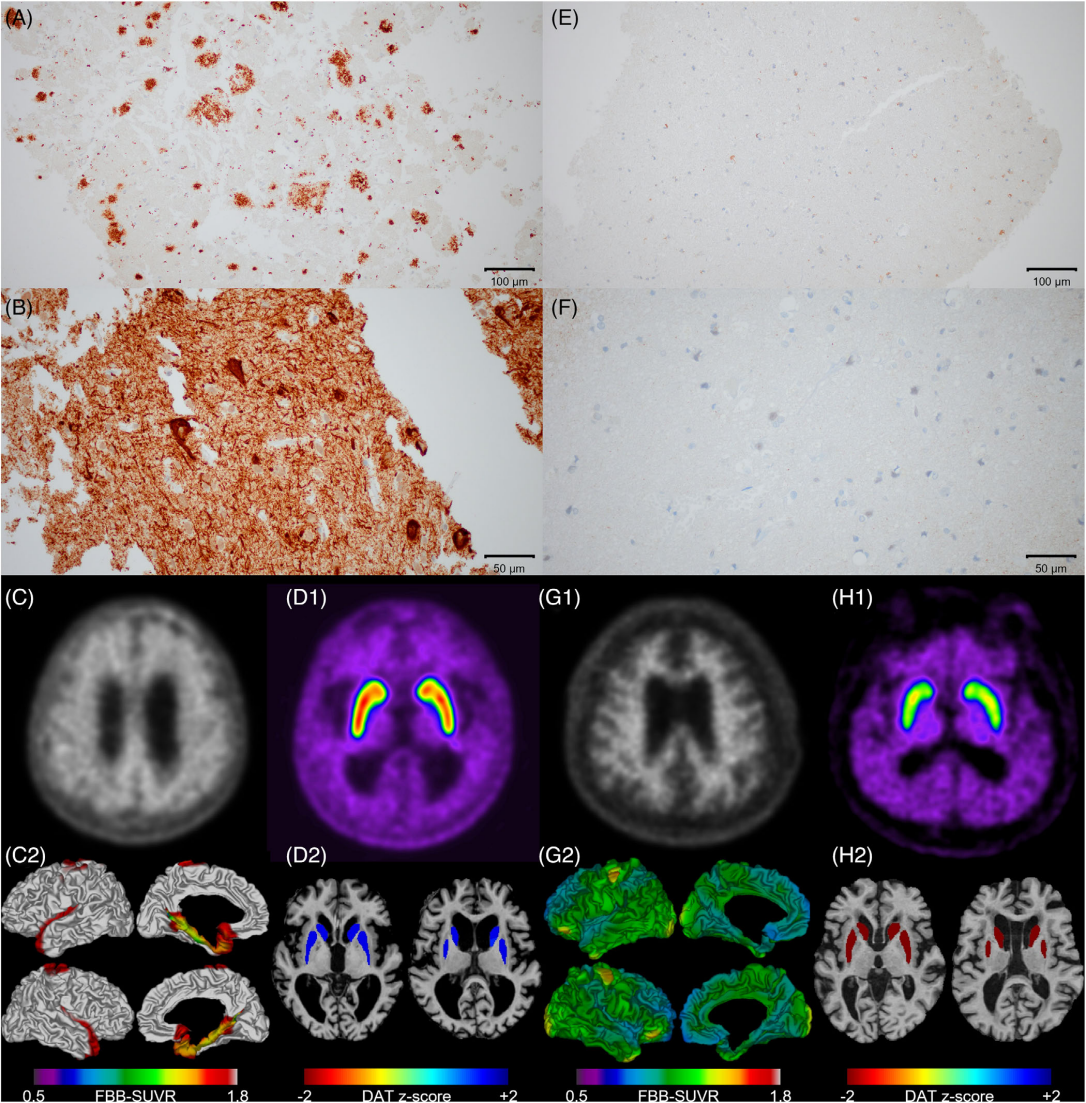
Immunohistochemistry findings for Aβ and phosphorylated tau on cortical biopsy samples, and FBB‐PET and DAT‐PET from two representative cases (Cases 1 and 35). Case 1 showed positive staining for Aβ (A, ×200) and phosphorylated tau (B, ×400) on cortical biopsy, with concordant Aβ positivity on the raw image (C1) and SUVR map (C2) of FBB‐PET, and normal DAT uptake on the raw image (D1) and SUVR z‐score map (D2) of DAT‐PET. This patient exhibited a poor clinical response to shunting. Case 35 demonstrated negative staining for Aβ (E, ×200) and phosphorylated tau (F, ×400), with concordant Aβ negativity on the raw image (G1) and SUVR map (G2) of FBB‐PET (G), and abnormally reduced DAT uptake on the raw image (H1) and SUVR z‐score map (H2) of DAT‐PET, with an excellent response to shunting. In the FBB SUVR maps, regions with SUVR values ≥ 1.478 (shown in red) were considered positive, whereas areas with higher Aβ deposition were depicted in gray. In the DAT‐PET SUVR z‐score maps, regions with SUVR values more than 1.5 standard deviations below those of healthy controls (as described in the Methods section) were shown in red. Scale bars: *A* and *E* = 100 µm; *B* and *F* = 50 µm. DAT, dopamine transporter; FBB, ^18^F‐florbetaben; PET, positron emission tomography; SUVR, standardized uptake value ratio.

Two of the 56 (3.6%) patients who underwent α‐synuclein staining had positive results: one of the 21 (4.8%) patients in the Aβ+ group and one of the 35 (2.9%) patients in the Aβ− group (*p* > 0.999). The two patients with positive α‐synuclein staining on biopsy did not undergo DAT‐PET imaging. Immunohistochemistry findings for α‐synuclein from one of these patients are shown in Figure S1. Two of the 56 (3.6%) patients who underwent TDP‐43 staining had positive results. Two of the 21 (9.5%) patients in the Aβ+ group were TDP‐43‐positive, while none in the 35 in the Aβ− group were (*p* = 0.136). Eleven of 57 (19.3%) patients who underwent pTau staining had positive results. Eleven of 22 (50.0%) patients in the Aβ+ group were pTau‐positive, while none in the 35 patients in the Aβ− group were (*p* < 0.001). Immunohistochemistry findings for Aβ and pTau on cortical biopsy samples, along with raw images of FBB‐PET and DAT‐PET from two representative cases, are shown in Figure 2. Among 56 patients who underwent GFAP staining, 51 (91.1%) showed GFAP positivity. Of the 35 Aβ− patients, 31 (88.6%) were GFAP‐positive, whereas 20 of the 21 Aβ+ patients (95.2%) were GFAP‐positive (*p* = 0.640).

### 3.2 Concordance between biopsy staining for Aβ and FBB‐PET results

Eleven of 21 (52.4%) patients who underwent FBB‐PET showed positive cerebral Aβ deposition. Eleven of 12 (91.7%) patients in the Aβ+ group had positive FBB‐PET results, while none of the nine patients in the Aβ− group did (*p* < 0.001). One patient who showed a discrepancy between biopsy Aβ staining and FBB‐PET findings had a borderline FBB‐SUVR (1.403). Single‐subject analysis revealed regional Aβ deposition in the left superior temporal cortex and bilateral medial prefrontal and medial parietal cortices (Figure S2). Of 21 who underwent FBB‐PET, 20 showed concordance with biopsy (accuracy: 95.2%, sensitivity: 91.7%, specificity: 100%).

### 3.3 Association between biopsy staining for Aβ and imaging findings

Thirty of 34 patients (88.2%) who underwent DAT‐PET showed abnormal DAT uptake. The proportions of patients with abnormal DAT uptake were not significantly different between the Aβ+ and Aβ− groups (11/14 [78.6%] vs 19/20 [95.0%], *p* = 0.283). Regional DAT uptake values were comparable between the Aβ+ and Aβ− groups in the anterior/posterior caudate and anterior/posterior putamen (Table 1).

The mean callosal angle, Evans index, ventriculomegaly score, dilated Sylvian fissure score, tight high convexity score, acute callosal angle score, focal sulcal dilatation score, and DESH scale were 72.56 ± 20.70, 0.37 ± 0.05, 1.65 ± 0.55, 1.04 ± 0.71, 1.06 ± 0.64, 1.67 ± 0.64, 0.63 ± 0.70, and 6.05 ± 1.79, respectively. The Aβ+ and Aβ− groups had comparable mean callosal angle (73.07 ± 16.88 vs 72.24 ± 23.04, *p* = 0.887) and Evans index (0.36 ± 0.04 vs 0.38 ± 0.06, *p* = 0.231). The ventriculomegaly score, dilated Sylvian fissure score, tight high convexity score, acute callosal angle score, focal sulcal dilatation score, and DESH scale were also comparable between the two groups.

### 3.4 Association between biopsy staining for Aβ and postoperative outcomes

**FIGURE 3:**
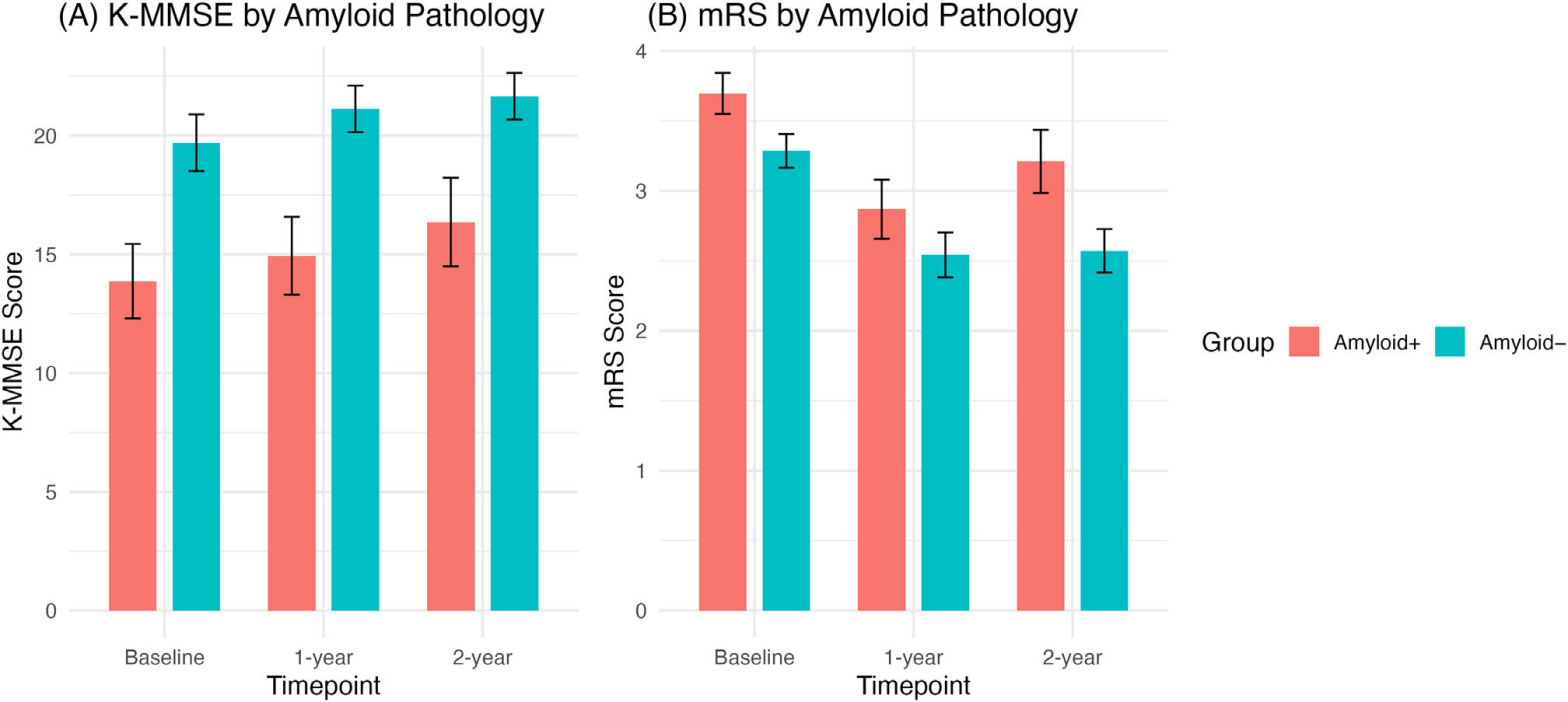
Longitudinal changes in cognitive and functional scores according to amyloid pathology. (A) K‐MMSE scores and (B) Modified Rankin Scale (mRS) scores at baseline, 1‐year, and 2‐year follow‐up in patients with positive (Aβ+, red) and negative (Aβ−, blue) cortical amyloid pathology on biopsy. Error bars indicate standard error of the mean. Higher K‐MMSE scores indicate better cognitive function, while lower mRS scores indicate better functional status. K‐MMSE, Korean Mini‐Mental State Examination.

Both Aβ+ and Aβ− groups showed improvement in K‐MMSE scores after surgery (Table 1 and Figure 3). In the Aβ+ group, baseline, 1‐year, and 2‐year postoperative K‐MMSE scores were 13.87 ± 7.51, 14.94 ± 7.84, and 15.27 ± 9.60, respectively. In the Aβ− group, the corresponding scores were 19.15 ± 7.63, 21.12 ± 5.78, and 21.65 ± 5.80. mRS improved in both groups at 1 year postoperatively, from 3.70 ± 0.70 to 2.87 ± 1.01 in the Aβ+ group, and from 3.29 ± 0.71 to 2.54 ± 0.95 in the Aβ− group. At 2 years, mRS remained improved in the Aβ− group (2.57 ± 0.92), whereas it worsened in the Aβ+ group (3.21 ± 1.08). Based on the LMMs, the total cohort exhibited a trend‐level improvement in MMSE at 1 year (*p* = 0.068), while the 2‐year improvement did not reach statistical significance (Table 2). In contrast, postoperative improvements in mRS were significant at both 1‐year and 2‐year follow‐ups. The Aβ− group demonstrated a significant improvement in MMSE at 1 year (*p* = 0.021) but not at 2 years (*p* = 0.123). In the Aβ+ group, neither 1‐year nor 2‐year MMSE changes were significant. For mRS, the Aβ− group showed significant improvement at both 1 and 2 years, while the Aβ+ group exhibited significant improvement at 1 year (*p* = 0.002) and a trend toward improvement at 2 years (*p* = 0.091).

**TABLE 2:** Longitudinal changes in MMSE and mRS according to Aβ staining.

	MMSE		mRS	
	Beta (SE)	*p* value	Beta (SE)	*p* value
Overall				
1‐year versus baseline	1.53 (0.82)	0.068	−0.78 (0.12)	<0.001
2‐year versus baseline	1.22 (1.07)	0.260	−0.61 (0.15)	<0.001
Aβ− subgroup				
1‐year versus baseline	2.33 (0.95)	0.021	−0.74 (0.13)	<0.001
2‐year versus baseline	1.92 (1.20)	0.123	−0.69 (0.17)	<0.001
Aβ+ subgroup				
1‐year versus baseline	0.18 (1.42)	0.903	−0.83 (0.23)	0.002
2‐year versus baseline	0.80 (2.05)	0.702	−0.50 (0.28)	0.091

*Note*: Linear mixed‐effects models with a random intercept for each participant were fitted to evaluate longitudinal changes in cognitive (MMSE) and functional (mRS) outcomes. Separate models were constructed for comparisons between baseline and 1‐year data and between baseline and 2‐year data. Each model included time (baseline vs follow‐up) as a fixed effect and was adjusted for age, sex, and education. Estimated marginal means (least‐square means) and pairwise contrasts were derived from the models. A positive estimate for MMSE indicates cognitive improvement, whereas a negative estimate for mRS indicates functional improvement.

Abbreviations: MMSE, Mini‐Mental State Examination; mRS, Modified Rankin Scale; SE, standard error.

### 3.5 Predictors of postoperative K‐MMSE scores

Among biopsy pathologies, positive staining for Aβ (beta [standard error, SE] = −3.38 [1.64], *p* = 0.046) and pTau (beta [SE] = −5.47 [1.89], *p* = 0.007) was associated with lower 1‐year postoperative K‐MMSE scores (Table 3). Positive staining for TDP‐43 showed a trend toward association with lower 1‐year K‐MMSE scores (beta [SE] = −9.45 [4.97], *p* = 0.066), while α‐synuclein‐positivity was not (beta [SE] = −1.34 [6.05], *p* = 0.825). Among MRI‐based iNPH indices, no significant predictor of 1‐year postoperative K‐MMSE score was identified. Regarding DAT uptake, reduced uptake in the anterior caudate (beta [SE] = −3.43 [1.25], *p* = 0.012) and anterior putamen (beta [SE] = −2.90 [1.21], *p* = 0.026) was significantly associated with better 1‐year postoperative K‐MMSE score, whereas posterior caudate and posterior putamen uptake were not.

**TABLE 3:** Predictors of postoperative K‐MMSE scores and mRS.

	1‐year K‐MMSE		2‐year K‐MMSE		1‐year mRS		2‐year mRS	
Predictor	Beta (SE)	*p* value	Beta (SE)	P value	Beta (SE)	*p* value	Beta (SE)	*p* value
Biopsy pathology								
Aβ	−3.38 (1.64)	0.046	−3.56 (2.01)	0.086	0.01 (0.26)	0.968	0.41 (0.30)	0.181
α‐Synuclein	−1.34 (6.05)	0.825	−5.06 (6.75)	0.460	−0.05 (0.67)	0.937	0.85 (0.70)	0.228
TDP‐43	−9.45 (4.97)	0.066	NA	NA	−0.11 (0.67)	0.867	1.02 (0.70)	0.153
pTau	−5.47 (1.89)	0.007	−8.62 (2.25)	0.001	0.23 (0.32)	0.478	0.72 (0.34)	0.038
GFAP	2.89 (2.71)	0.295	2.54 (3.14)	0.425	−0.08 (0.44)	0.854	0.38 (0.48)	0.427
MRI findings								
Callosal angle	0.004 (0.04)	0.919	0.01 (0.05)	0.789	4.07 × 10^−4^ (0.01)	0.947	2.08 × 10^−4^ (0.01)	0.978
Evans index	9.57 (16.05)	0.555	6.84 (20.68)	0.743	1.29 (2.58)	0.620	2.05 (3.11)	0.513
Ventriculomegaly score	0.55 (1.50)	0.718	1.44 (2.22)	0.521	0.26 (0.22)	0.245	−0.06 (0.33)	0.849
Dilated Sylvian fissure score	−0.88 (1.13)	0.440	−1.72 (1.54)	0.272	0.06 (0.19)	0.751	0.11 (0.22)	0.629
Tight high convexity score	1.80 (1.15)	0.125	1.92 (1.58)	0.233	−0.25 (0.20)	0.217	−0.27 (0.23)	0.248
Acute callosal angle score	−0.36 (1.20)	0.765	−0.49 (1.47)	0.742	−0.12 (0.20)	0.548	−0.11 (0.26)	0.670
Focal sulcal dilatation score	0.13 (1.37)	0.927	−0.36 (1.61)	0.826	0.11 (0.21)	0.602	−0.11 (0.23)	0.642
DESH scale	0.16 (0.44)	0.712	−0.02 (0.65)	0.980	3.86 × 10^−4^ (0.07)	0.996	−0.05 (0.09)	0.539
DAT uptake values								
Anterior caudate	−3.43 (1.25)	0.012	−5.37 (1.48)	0.002	0.12 (0.22)	0.600	0.62 (0.22)	0.011
Posterior caudate	−2.73 (1.45)	0.074	−5.29 (1.77)	0.008	0.16 (0.24)	0.507	0.57 (0.24)	0.031
Anterior putamen	−2.90 (1.21)	0.026	−4.85 (1.52)	0.005	0.12 (0.21)	0.567	0.55 (0.21)	0.017
Posterior putamen	−1.81 (1.22)	0.152	−3.72 (1.61)	0.034	0.30 (0.18)	0.118	0.49 (0.19)	0.018

*Note*: Data represent results from general linear models for postoperative K‐MMSE score or mRS, adjusted for age, sex, education, and baseline score.

Abbreviations: DAT, dopamine transporter; DESH, disproportionately enlarged subarachnoid space hydrocephalus; K‐MMSE, Korean‐version Mini‐Mental State Examination; MRI, magnetic resonance imaging; NA, not applicable due to small sample size; TDP‐43, TAR DNA‐binding protein 43.

For 2‐year postoperative K‐MMSE scores, only pTau‐positivity remained a significant biopsy‐related predictor (beta [SE] = −8.62 [2.25], *p* = 0.001); Aβ, α‐synuclein, and TDP‐43 were not associated. Similarly, MRI‐based iNPH indices were not predictive. In contrast, reduced DAT uptake across all striatal regions was significantly associated with better 2‐year K‐MMSE scores.

### 3.6 Predictors of postoperative mRS

None of the biopsy pathologies, MRI‐based iNPH indices, or regional DAT uptake values were significantly associated with 1‐year postoperative mRS (Table 3). For 2‐year postoperative mRS, positive pTau staining was associated with worse mRS (beta [SE] = 0.72 [0.34], *p* = 0.038), while Aβ, α‐synuclein, and TDP‐43 were not. No MRI‐based iNPH indices were predictive of 2‐year mRS. Reduced DAT uptake across all striatal regions was significantly associated with better 2‐year mRS.

### 3.7 Interaction between Aβ pathology and DAT uptake on postoperative prognosis

When the interaction terms between biopsy‐confirmed Aβ pathology and anterior caudate DAT uptake value were tested, a significant interaction effect was observed on 1‐year postoperative K‐MMSE scores, with a trend toward significance at 2 years (Table 4). Subgroup analyses revealed that lower anterior caudate DAT uptake was associated with better 1‐ and 2‐year K‐MMSE scores in the Aβ+ subgroup, but not in the Aβ− subgroup. In contrast, lower anterior caudate DAT uptake was associated with better 2‐year mRS in both subgroups, although statistical significance was reached only in the Aβ+ subgroup. Similarly, when the interaction terms between biopsy‐confirmed pTau pathology and anterior caudate DAT uptake were tested, significant interaction effects were found on 1‐ and 2‐year postoperative K‐MMSE scores. However, subgroup analyses did not reveal significant associations between anterior caudate DAT uptake and 1‐ and 2‐year K‐MMSE scores or mRS outcomes.

**TABLE 4:** Interaction effects of biopsy pathologies and anterior caudate DAT uptake on postoperative prognosis.

		1‐year K‐MMSE		2‐year K‐MMSE		1‐year mRS		2‐year mRS	
Subject	Predictor	Beta (SE)	*p* value	Beta (SE)	*p* value	Beta (SE)	*p* value	Beta (SE)	*p* value
Overall	Aβ × DAT uptake	−5.58 (2.05)	0.014	−5.41 (2.95)	0.086	0.17 (0.43)	0.687	0.23 (0.40)	0.568
Aβ− subgroup	DAT uptake	2.22 (2.56)	0.409	−0.50 (2.71)	0.861	0.09 (0.59)	0.878	0.43 (0.56)	0.469
Aβ+ subgroup	DAT uptake	−5.08 (1.33)	0.009	−6.23 (2.06)	0.023	0.19 (0.25)	0.480	0.84 (0.23)	0.014
Overall	pTau × DAT uptake	−5.17 (1.89)	0.014	−5.72 (2.23)	0.022	0.12 (0.41)	0.776	0.36 (0.40)	0.379
pTau− subgroup	DAT uptake	1.35 (1.93)	0.497	−0.39 (2.17)	0.863	0.19 (0.46)	0.680	0.27 (0.45)	0.556
pTau+ subgroup	DAT uptake	NA	NA	−6.60 (2.23)	0.207	0.04 (0.21)	0.888	0.97 (0.19)	0.124

*Note*: Data represent results from general linear models for postoperative K‐MMSE score or mRS, adjusted for age, sex, education, and baseline scores. For the overall cohort, the interaction term between Aβ staining and caudate DAT uptake was used as a predictor, adjusted for age, sex, education, baseline score, Aβ positivity, and caudate DAT uptake. For analyses within the Aβ+ and Aβ− subgroups, caudate DAT uptake was used as a predictor, adjusting for age, sex, education, and baseline score.

Abbreviations: DAT, dopamine transporter; K‐MMSE, Korean Mini‐Mental State Examination; mRS, Modified Rankin Scale; NA, not applicable due to small sample size; SE, standard error.

## 4 DISCUSSION

In this study, we investigated the efficacy and prognostic factors of VP shunting in patients with iNPH, whose degenerative pathologies were identified through cortical biopsy and imaging biomarkers. We also evaluated the concordance between biopsy‐identified Aβ pathology and amyloid PET results. Our major findings are as follows. First, 39.7% of iNPH patients exhibited biopsy‐confirmed Aβ pathology. Aβ‐positivity was associated with worse 1‐year K‐MMSE scores, whereas positive pTau staining was linked to poorer 1‐ and 2‐year K‐MMSE scores as well as 2‐year mRS outcomes. Second, regardless of the presence of Aβ pathology, improvements in mRS were observed, although the mRS improvement in Aβ+ patients decreased to a tendency‐level significance at the 2‐year follow‐up. Third, the concordance rate between biopsy‐confirmed Aβ pathology and amyloid PET positivity was 20 out of 21 cases (95.2%); the one discordant case showed regional Aβ deposition on a voxel‐wise PET analysis. Fourth, lower DAT uptake in the anterior striatum was associated with better 1‐year K‐MMSE, with this beneficial effect being more prominent in Aβ+ and pTau+ patients. Lower DAT uptake in the overall striatum was additionally associated with better 2‐year K‐MMSE and mRS outcomes. Taken together, our results suggest that VP shunting is an effective treatment for iNPH, and cortical biopsy performed during the procedure can provide information on Aβ pathology comparable to that of amyloid PET. Although Aβ pathology is associated with relatively poorer postoperative outcomes, VP shunt remains effective even in Aβ+ patients, particularly when dopaminergic degeneration of the anterior striatum is present.

About 40% of iNPH patients exhibited Aβ pathology on cortical biopsy. Aβ positivity was associated with lower 1‐year K‐MMSE scores, while positive pTau staining was linked to poorer K‐MMSE scores at both 1 and 2 years, as well as worse 2‐year mRS outcomes. These findings are consistent with previous studies utilizing cortical biopsy in iNPH patients, which reported AD‐related neuropathological changes in 18% to 50% of cases.^27^, ^28^, ^29^, ^30^ This supports the notion that AD is a prevalent pathological comorbidity in iNPH.^14^ The adverse impact of Aβ and pTau positivity on cognition and daily functioning observed in our study further underscores the prognostic relevance of AD pathology in iNPH. Previous investigations into the effect of biopsy‐confirmed AD pathology on surgical outcomes have shown inconsistent results – some reported significant detrimental effects,^31^, ^32^ while others found no significant association,^27^, ^28^, ^33^ possibly due to differences in cohort composition (e.g., the prevalence of concomitant LBD pathology) and variations in the methodologies used to assess cognitive and functional outcomes. Our findings suggest that AD pathology contributes to postoperative outcomes in iNPH, possibly via mechanisms consistent with the amyloid‐to‐tau cascade model of AD.^34^ However, while Aβ pathology had a significant negative effect on 1‐year MMSE, it did not significantly affect mRS outcomes (Table 3). Moreover, improvements in mRS were observed regardless of Aβ status in our iNPH patients, although in Aβ+ patients the significance declined to a trend level at 2 years. Given the progressive cognitive and functional decline typically seen in AD, it is plausible that, without shunt surgery, Aβ+ patients might not have simply shown less improvement, but rather experienced accelerated deterioration. Although this hypothesis requires validation in clinical trials including a non‐surgical control arm,^35^ iNPH patients could benefit from VP shunt regardless of Aβ positivity.^27^

The high concordance between Aβ positivity on biopsy and amyloid PET results supports the utility of cortical biopsy as a reliable indicator of cerebral amyloid deposition. Notably, 11 of 12 Aβ+ patients who underwent FBB‐PET were confirmed as PET‐positive, and the remaining patient exhibited regional amyloid deposition on a voxel‐wise analysis. These results validate the relevance of intraoperative biopsy in identifying underlying AD pathology, consistent with prior studies reporting significant association between Aβ load in the right frontal cortical biopsy and the right frontal cortex and global amyloid PET uptake values in patients with iNPH.^36^ In the Aβ+ group, 50% showed pTau positivity, whereas no pTau positivity was observed in the Aβ− group. Considering that the biopsy site – the frontal cortex – is a region where tau pathology is typically observed in Braak stage V–VI,^37^, ^38^, ^39^ this finding supports the view that cortical biopsy can reliably reflect the staging of AD pathology. In contrast, α‐synuclein and TDP‐43 pathologies were observed in only two cases each. Both cases that showed positive TDP‐43 were also positive for Aβ and pTau, suggesting that the TDP‐43 pathology in these patients is likely comorbid with AD, which may correspond to stage V in the five‐stage TDP‐43 staging system proposed by Josephs et al.^40^ Among the two patients with α‐synuclein positivity, one was also positive for Aβ and pTau, while the other was negative for both. Given the high clinical diagnostic rate of DLB or pLBD and the frequent abnormal DAT‐PET findings in our iNPH cohort, these results suggest that cortical biopsy may not be sufficiently sensitive for the detection of LBD pathology.

**FIGURE 4:**
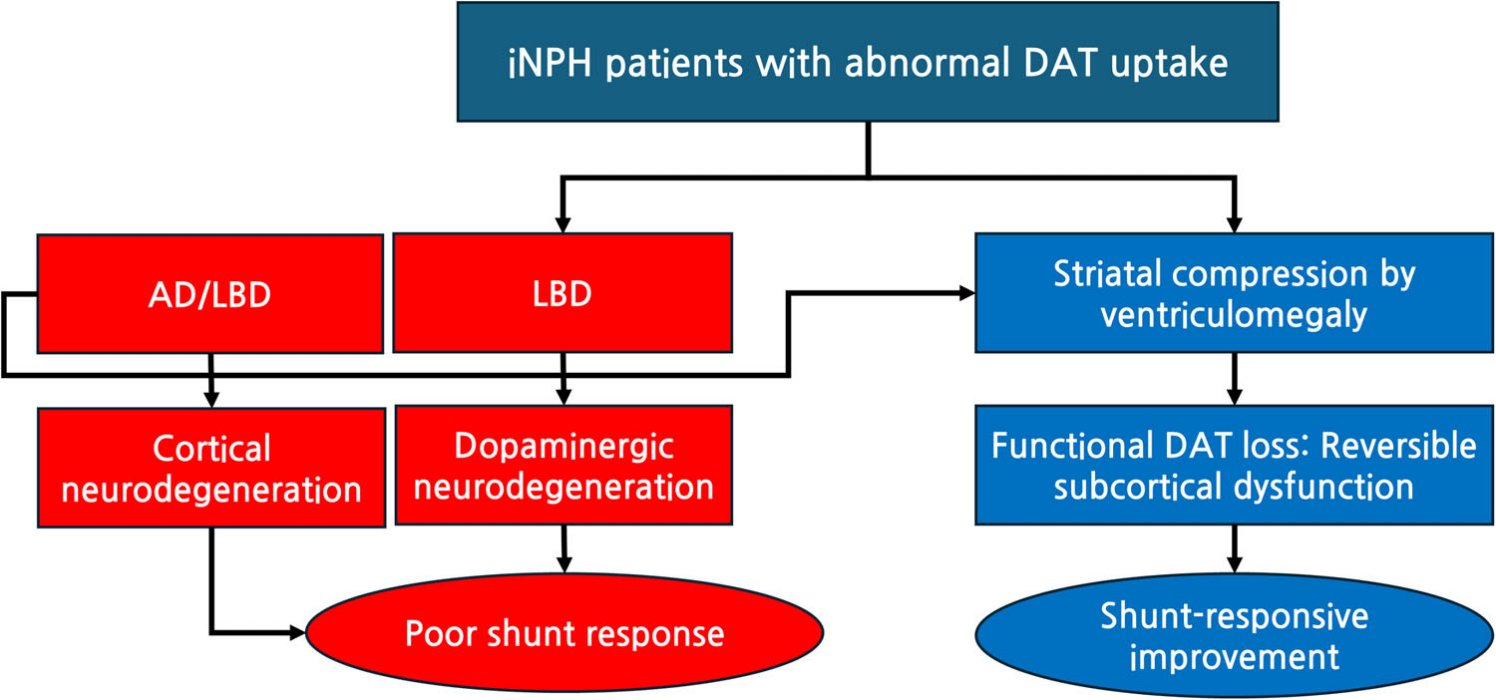
Conceptual model illustrating two possible mechanisms underlying abnormal DAT uptake in iNPH. Left pathway: coexisting Lewy body disease (LBD) or cortical neurodegeneration (e.g., AD/LBD) may result in irreversible dopaminergic or cortical damage, leading to poor shunt response. Right pathway: striatal compression due to ventriculomegaly may cause functional DAT loss, representing a reversible subcortical dysfunction that is responsive to CSF diversion. AD, Alzheimer's disease; CSF, cerebrospinal fluid; DAT, dopamine transporter.

Striatal dopaminergic depletion, especially in the anterior striatum as assessed by DAT‐PET, was paradoxically associated with better cognitive and functional outcomes. This counterintuitive finding may reflect a subset of iNPH patients whose cognitive impairment is driven less by cortical neurodegeneration and more by subcortical dysfunction that is responsive to CSF diversion. iNPH shares clinical and imaging features, such as gait disturbance, cognitive decline, and anterior striatal DAT abnormalities,^9^, ^41^, ^42^ with LBD, particularly DLB. iNPH may contribute to the progression of LBD by compressing dopaminergic pathways through ventricular enlargement – an effect that may be alleviated by shunt surgery, potentially leading to better treatment response.^9^, ^43^ To visually summarize this hypothesis, we included a conceptual schematic (Figure 4) distinguishing two possible mechanisms underlying abnormal DAT uptake in iNPH: one reflecting irreversible dopaminergic neurodegeneration and another reflecting reversible subcortical dysfunction. The latter may be particularly responsive to CSF diversion therapy, offering a potential explanation for the observed improvement in patients with reduced anterior striatal DAT uptake.

While patients in the Aβ− group exhibited postoperative improvement regardless of the degree of DAT depletion, those in the Aβ+ group showed better outcomes when anterior striatal DAT depletion was more severe. Anterior striatal DAT depletion is most frequently observed pattern in iNPH^8^, ^42^ and is associated with Aβ deposition, spanning from healthy aging to cognitive impairment across the AD–LBD spectrum, as demonstrated in our previous study.^44^ In that study, anterior striatal DAT depletion and Aβ deposition interacted negatively to influence cognition: The degree of cognitive dysfunction in patients with abnormalies in both PET scans (high global FBB SUVR and low anterior striatal DAT uptake) was smaller than expected if the abnormalities in each PET scan contributed independently and additively to cognitive dysfunction. This finding supports the brain reserve hypothesis,^45^ suggesting that patients with greater LBD‐related DAT depletion (reflected in the anterior striatal DAT uptake) may have a relatively lower AD pathological burden required to produce a similar degree of cognitive impairment. Consistent with this interpretation, anterior caudate (*ρ* = 0.842, *p* = 0.074, *N* = 5) and anterior putamen (*ρ* = 0.853, *p* = 0.066, *N* = 5) DAT uptake values showed trend‐level positive correlations with global FBB SUVR in the Aβ+ subgroup. Collectively, these findings suggest that the anterior striatum serves as a critical hub where the interaction between AD and LBD pathologies converges and interacts with mechanical compression in iNPH.

This study has several limitations. First, it was conducted retrospectively at a single tertiary referral center, which might have introduced selection bias and limited the generalizability of our findings to broader iNPH populations. Second, the sample sizes for amyloid PET and DAT‐PET subgroups were relatively small, potentially reducing statistical power and increasing the risk of type II error in some of the subgroup analyses. Third, cortical biopsy specimens were obtained exclusively from the frontal cortex, which may not fully represent the regional heterogeneity of neurodegenerative pathologies such as LBD and TDP‐43 proteinopathies. The limited detection of α‐synuclein and TDP‐43 in our cohort may partly reflect this sampling constraint. In particular, α‐synuclein pathology in LBD often begins in the brainstem or limbic structures before spreading to the neocortex, and TDP‐43 pathology typically follows a sequential pattern involving the medial temporal lobe. Therefore, sampling from a single neocortical region may substantially underestimate the true burden of these pathologies. Future studies employing multisite sampling or advanced in vivo biomarkers will be needed to improve pathological detection and characterization. Fourth, the lack of a non‐surgical control group precludes a definitive evaluation of whether the observed postoperative improvements, particularly in patients with concomitant AD pathology, exceed the natural disease course. Fifth, although a standardized clinical approach was used to diagnose comorbid neurodegenerative conditions, diagnostic misclassification cannot be entirely excluded, especially given the overlapping clinical features of iNPH and LBD. Sixth, while DAT‐PET findings revealed paradoxical associations with cognitive outcomes, the interpretation of these results remains speculative, and the underlying mechanisms require further clarification. Seventh, of the 34 patients who underwent DAT‐PET, quantitative analysis could not be performed in six patients due to difficulties in image processing, and 10 patients underwent DAT‐PET after the shunt surgery. Although further adjusting for the interval between DAT imaging and shunt surgery did not alter the results, future studies comparing DAT‐PET findings before and after shunt placement are warranted. Finally, cognitive function was assessed using only the K‐MMSE, and neither the Montreal Cognitive Assessment nor detailed neuropsychological testing was consistently administered after shunt surgery. This limits our ability to detect domain‐specific cognitive changes or more subtle postoperative improvements. In addition, real‐world functional outcomes such as gait speed, continence, and domain‐specific activities of daily living were not systematically assessed. Although the mRS was used to estimate overall functional status, it may not adequately reflect more granular changes in everyday functioning. This limits the interpretability of postoperative improvements and may reduce the generalizability of our findings to broader clinical contexts.

In conclusion, our results suggest that VP shunting remains beneficial in iNPH patients even in the presence of underlying degenerative comorbidities and that dopaminergic imaging may serve as a useful prognostic marker in this population. Cortical biopsy may also provide a valuable means of detecting comorbid AD pathology, thereby facilitating more accurate diagnosis and prognostication.

## CONFLICT OF INTEREST STATEMENT

The authors have no interests to disclose. All author disclosures are available in Supporting Information.

## CONSENT STATEMENT

The need for informed consent was waived due to the retrospective nature of the study.

## Supporting information



Supporting Information

Supporting Information
