# Supplementary material for: Degenerative pathologies on cortical biopsy, dopaminergic depletion, and shunt efficacy in iNPH
Source: Alzheimers Dement. 2025 Dec 9;21(12):e70974. doi: 10.1002/alz.70974 (PMC12689450; doi:10.1002/alz.70974)
Supplement: Supplementary file 1 — Supporting Information [file ALZ-21-e70974-s001.docx]

**Supplementary Table 1.** Clinical, cortical biopsy, amyloid PET, and CSF biomarker information of individual study participants

|  |  |  |  | Cortical biopsy staining | | | |  |  | CSF | MMSE | | | mRS | | |
| --- | --- | --- | --- | --- | --- | --- | --- | --- | --- | --- | --- | --- | --- | --- | --- | --- |
| Case No | Age | Sex | Concomitant clinical diagnosis | Aβ | Tau | TDP-43 | α-syn | *APOE4* | Amyloid PET result | SAA | Preop | Postop 1yr | Postop 2yr | Preop | Postop 1yr | Postop 2yr |
| 1 | 61 | F | AD with LBD | **Positive** | **Positive** | Negative | Negative | Carrier | **Positive** | Not done | 11 | 4 | NA | 3 | 3 | 4 |
| 2 | 81 | M | MCI d/t LBD | Negative | Negative | Negative | Negative | Non-carrier | Negative | Not done | 25 | 27 | 23 | 4 | 2 | 3 |
| 3 | 71 | M | DLB | **Positive** | Negative | Negative | Negative | Non-carrier | Not done | Not done | 14 | 20 | 26 | 4 | 3 | 4 |
| 4 | 79 | F | DLB | **Positive** | Negative | Negative | Negative | Non-carrier | Not done | Not done | 22 | 22 | 19 | 4 | 3 | 3 |
| 5 | 88 | M | DLB | **Positive** | Negative | Negative | Negative | Carrier | Not done | Not done | 5 | 13 | NA | 4 | 3 | NA |
| 6 | 76 | M | DLB | Negative | Negative | Negative | Negative | Non-carrier | Not done | Not done | 13 | 19 | 20 | 3 | 2 | 2 |
| 7 | 67 | M | DLB | Negative | Negative | Negative | Negative | Non-carrier | Not done | Not done | 24 | 29 | 26 | 3 | 1 | 1 |
| 8 | 74 | F | AD with DLB | **Positive** | **Positive** | Negative | Negative | Carrier | Not done | Not done | 4 | 0 | 0 | 4 | 3 | 5 |
| 9 | 56 | M | DLB | Negative | Negative | Negative | Negative | Non-carrier | Negative | Not done | 17 | 15 | 20 | 2 | 3 | 3 |
| 10 | 82 | M | AD with DLB | **Positive** | **Positive** | Negative | Negative | Non-carrier | Negative | Not done | 22 | 19 | 17 | 4 | 2 | 3 |
| 11 | 84 | F | AD with Vascular dementia | Negative | Negative | Negative | Negative | Non-carrier | Not done | Not done | 17 | 14 | 11 | 3 | 4 | 4 |
| 12 | 78 | F | AD with DLB | **Positive** | **Positive** | **Positive** | **Positive** | Non-carrier | **Positive** | Not done | 10 | NA | NA | 4 | 3 | 5 |
| 13 | 48 | F | MCI | Negative | Negative | Negative | Negative | Non-carrier | Not done | Not done | 30 | NA | 30 | 3 | 3 | 4 |
| 14 | 79 | M | LBD | Negative | Negative | Negative | Negative | Non-carrier | Negative | Not done | 7 | 20 | 24 | 4 | 3 | 3 |
| 15 | 88 | F | MCI d/t AD with DLB | **Positive** | **Positive** | Negative | Negative | Non-carrier | **Positive** | Not done | 21 | NA | NA | 3 | 5 | 5 |
| 16 | 83 | M | AD with LBD | **Positive** | **Positive** | **Positive** | Negative | Non-carrier | **Positive** | Not done | 10 | 5 | NS | 4 | 3 | 3 |
| 17 | 53 | F | MCI | Negative | Negative | Negative | Negative | Non-carrier | Not done | Not done | 27 | 29 | 30 | 3 | 2 | 2 |
| 18 | 73 | F | Old thalamic ICH | Negative | Negative | Negative | Negative | Carrier | Not done | Not done | NA | NA | NA | 4 | 3 | 3 |
| 19 | 80 | M | DLB with amyloid deposition | **Positive** | Negative | Negative | Negative | Non-carrier | **Positive** | Not done | 13 | 11 | 23 | 4 | 2 | 2 |
| 20 | 76 | F | LBD | Negative | Negative | Negative | Negative | Non-carrier | Negative | Not done | 21 | 23 | 23 | 4 | 3 | 3 |
| 21 | 50 | M | MCI d/t LBD | Negative | Negative | Negative | **Positive** | Non-carrier | Not done | Not done | 28 | 30 | 27 | 3 | 2 | 2 |
| 22 | 82 | M | AD | **Positive** | **Positive** | Negative | Negative | Carrier | **Positive** | Not done | 26 | 22 | 19 | 3 | 2 | 2 |
| 23 | 77 | F | DLB with regional amyloid AD | **Positive** | Negative | Negative | Negative | Non-carrier | Negative (**regional positive**: cingulum and left superior temporal) | Not done | 4 | 6 | NA | 4 | 2 | 4 |
| 24 | 83 | F | AD with DLB | Negative | Negative | Negative | Negative | Non-carrier | Not done | Not done | 18 | 19 | 15 | 3 | 2 | 3 |
| 25 | 80 | M | LBD | Negative | Negative | Negative | Negative | Non-carrier | Not done | Not done | 23 | 24 | 25 | 3 | 1 | 1 |
| 26 | 75 | F | Pure NPH | Negative | Negative | Negative | Negative | Non-carrier | Not done | Not done | 30 | NA | 28 | 4 | 3 | 2 |
| 27 | 72 | F | LBD with Vascular dementia | Negative | Negative | Negative | Negative | Non-carrier | Not done | Not done | 12 | 11 | 9 | 4 | 4 | 4 |
| 28 | 79 | F | MCI d/t DLB | **Positive** | Negative | Negative | Negative | Non-carrier | Not done | Not done | 24 | 20 | 21 | 2 | 3 | 3 |
| 29 | 71 | F | DLB | Negative | Negative | Negative | Negative | Non-carrier | Not done | Not done | 17 | 24 | 22 | 4 | 3 | 4 |
| 30 | 73 | F | LBD | Negative | Negative | Negative | Negative | NA | Not done | Not done | 26 | NA | NA | 2 | 1 | 1 |
| 31 | 72 | F | Pure NPH | Negative | Negative | Negative | Negative | Non-carrier | Not done | Not done | 12 | 16 | 17 | 2 | 1 | 2 |
| 32 | 80 | F | AD with DLB | **Positive** | Negative | Negative | Negative | Carrier | **Positive** | Negative | 15 | 17 | 11 | 2 | 1 | 2 |
| 33 | 71 | F | DLB | Negative | Negative | Negative | Negative | Non-carrier | Not done | Not done | 21 | 21 | 21 | 2 | 1 | 2 |
| 34 | 82 | M | DLB with regional amyloid | Negative | Negative | Negative | Negative | Carrier | Negative | Not done | 15 | 17 | NA | 3 | 3 | 2 |
| 35 | 65 | M | DLB | Negative | Negative | Negative | Negative | Non-carrier | Negative (**regional positive**: left temporo-parietal) | Negative | 10 | 23 | 26 | 4 | 3 | 2 |
| 36 | 61 | F | DLB with AD | **Positive** | **Positive** | Negative | Negative | Non-carrier | **Positive** | Negative | 0 | NA | 0 | 4 | 2 | 3 |
| 37 | 70 | M | Pure NPH | Negative | Negative | Negative | Negative | NA | Not done | Not done | 25 | NA | NA | 3 | 2 | 2 |
| 38 | 67 | F | MCI | **Positive** | Negative | Negative | Negative | NA | Not done | Not done | 16 | NS | 20 | 5 | 3 | 2 |
| 39 | 76 | F | LBD | Negative | Negative | Negative | Negative | Non-carrier | Not done | Negative | 16 | 20 | 15 | 3 | 2 | NA |
| 40 | 74 | F | MCI d/t DLB | **Positive** | **Positive** | Negative | Negative | Carrier | Not done | Not done | 11 | 23 | 24 | 4 | 3 | 2 |
| 41 | 71 | M | Pure NPH | Negative | Negative | Negative | Negative | NA | Not done | Not done | 5 | NA | NA | 4 | 3 | NA |
| 42 | 76 | M | MCI d/t DLB | Negative | Negative | Negative | Negative | Non-carrier | Negative | Negative | 26 | 26 | 22 | 3 | 3 | 3 |
| 43 | 75 | F | Pure NPH | Negative | Negative | Negative | Negative | NA | Not done | Not done | 23 | NA | NA | 3 | 2 | 2 |
| 44 | 79 | M | MCI d/t DLB | Negative | Negative | Negative | Negative | Non-carrier | Not done | **Positive** | 28 | 27 | 24 | 4 | 2 | 2 |
| 45 | 77 | M | AD | **Positive** | Negative | Negative | Negative | NA | **Positive** | Not done | 25 | 23 | 24 | 4 | 2 | 2 |
| 46 | 77 | M | DLB with AD | **Positive** | NA | NA | NA | Non-carrier | **Positive** | Not done | 15 | 23 | 21 | 4 | 3 | NA |
| 47 | 78 | F | DLB | **Positive** | **Positive** | NA | NA | Non-carrier | Not done | Not done | 18 | 11 | 4 | 4 | 3 | 4 |
| 48 | 73 | F | MCI d/t DLB | Negative | Negative | Negative | Negative | Non-carrier | Not done | Negative | 25 | 20 | 26 | 3 | 2 | 3 |
| 49 | 64 | M | Pure NPH | Negative | Negative | Negative | Negative | Non-carrier | Not done | Not done | 28 | NA | NA | 3 | 3 | NA |
| 50 | 67 | F | DLB | Negative | Negative | Negative | Negative | Non-carrier | Not done | Not done | 13 | 23 | NA | 3 | 2 | NA |
| 51 | 81 | M | AD | Negative | Negative | Negative | Negative | Non-carrier | Not done | Not done | 14 | NA | NA | 4 | 4 | NA |
| 52 | 77 | M | LBD with AD | **Positive** | Negative | Negative | Negative | Non-carrier | Not done | Not done | 15 | NA | NA | 4 | 5 | NA |
| 53 | 85 | F | DLB | Negative | Negative | Negative | Negative | Non-carrier | Negative | Not done | 13 | 8 | 14 | 3 | 4 | 3 |
| 54 | 74 | F | MCI d/t DLB | Negative | Negative | Negative | Negative | Non-carrier | Not done | Negative | 27 | 27 | NA | 4 | 4 | 4 |
| 55 | 72 | F | Pure NPH | Negative | Negative | Negative | Negative | NA | Not done | Not done | NA | NA | NA | 3 | 2 | NA |
| 56 | 70 | M | Pure NPH | **Positive** | Negative | Negative | Negative | NA | Not done | Not done | 16 | NA | NA | 3 | 2 | 3 |
| 57 | 77 | F | Pure NPH | **Positive** | **Positive** | Negative | Negative | NA | Not done | Not done | 2 | NA | NA | 4 | 5 | NA |
| 58 | 80 | F | DLB with Vascular dementia | Negative | Negative | Negative | Negative | Non-carrier | Negative | Not done | 14 | 16 | NA | 5 | 4 | NA |

Abbreviations: AD, Alzheimer’s disease; *APOE4, apolipoprotein ε4*; α-syn, α-synuclein; DLB, dementia with Lewy bodies; ICH, intracranial hemorrhage; LBD, Lewy body disease; MCI, mild cognitive impairment; MMSE, Mini-Mental State Examination; mRS, modified Rankin Scale; NA, not applicable; NPH, normal pressure hydrocephalus; PET, positron emission tomography. Abnormal biomarker results are expressed in bold.


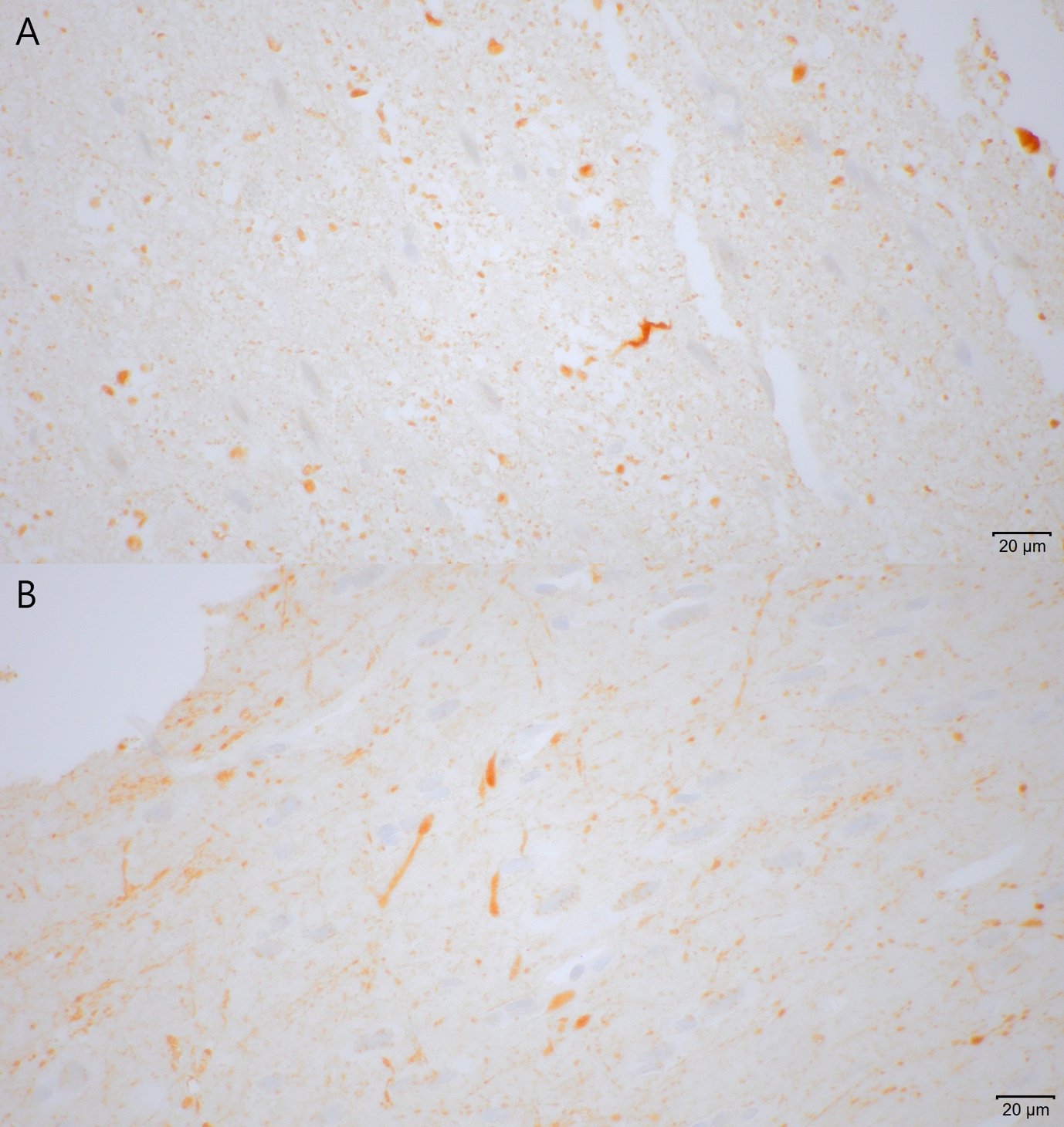


**Supplementary Figure 1.** Immunohistochemistry findings for α-synuclein in cortical biopsy samples. Representative sections from a patient with positive α-synuclein staining. Original magnification: ×600. Scale bars: A and B = 20 μm.


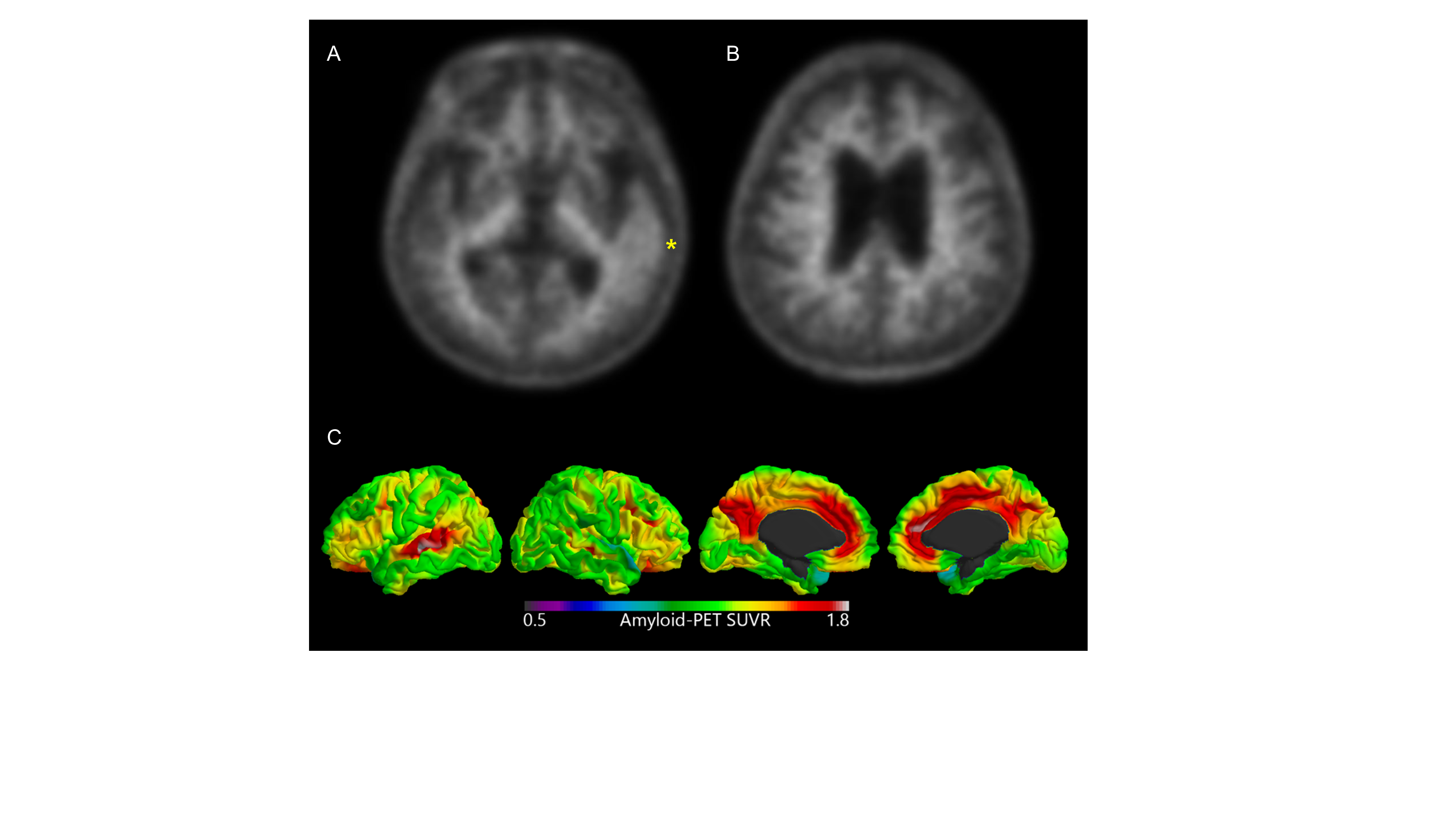


**Supplementary Figure 2.** Raw images and single-subject analysis results from the patient showing a discrepancy between cortical biopsy Aβ staining and FBB-PET positivity. This patient showed positive Aβ staining on cortical biopsy, but the global FBB-SUVR was 1.403, which is below the cut-off value of 1.478 for amyloid positivity. Voxel-wise PET analysis revealed focal amyloid deposition on the SUVR map in the bilateral medial prefrontal, precuneus, and right lateral temporal regions. Regions with SUVR values ≥ 1.478 (shown in red) were considered positive, whereas areas with higher Aβ deposition were depicted in gray. An asterisk indicates visually detectable regional Aβ deposition in the left temporal cortex.
